# IL-6 and IL-8: An Overview of Their Roles in Healthy and Pathological Pregnancies

**DOI:** 10.3390/ijms232314574

**Published:** 2022-11-23

**Authors:** Aleksandra Vilotić, Mirjana Nacka-Aleksić, Andrea Pirković, Žanka Bojić-Trbojević, Dragana Dekanski, Milica Jovanović Krivokuća

**Affiliations:** Department for Biology of Reproduction, Institute for Application of Nuclear Energy (INEP), University of Belgrade, Banatska 31b, 11080 Belgrade, Serbia

**Keywords:** IL-6, IL-8, inflammation, pregnancy, pregnancy complications

## Abstract

Interleukin-6 (IL-6) is an acknowledged inflammatory cytokine with a pleiotropic action, mediating innate and adaptive immunity and multiple physiological processes, including protective and regenerative ones. IL-8 is a pro-inflammatory CXC chemokine with a primary function in attracting and activating neutrophils, but also implicated in a variety of other cellular processes. These two ILs are abundantly expressed at the feto-maternal interface over the course of a pregnancy and have been shown to participate in numerous pregnancy-related events. In this review, we summarize the literature data regarding their role in healthy and pathological pregnancies. The general information related to IL-6 and IL-8 functions is followed by an overview of their overall expression in cycling endometrium and at the feto-maternal interface. Further, we provide an overview of their involvement in pregnancy establishment and parturition. Finally, the implication of IL-6 and IL-8 in pregnancy-associated pathological conditions, such as pregnancy loss, preeclampsia, gestational diabetes mellitus and infection/inflammation is discussed.

## 1. Introduction

The initial step in the establishment of a pregnancy is the implantation of an dembryo at the blastocyst stage into the receptive endometrium (decidua). This is followed by the formation of the placenta, a transient organ essential for fetal development and growth [1]. The development of the placenta depends on the differentiation of the cells from the outer layer of the blastocyst, i.e., trophectoderm, into specialized trophoblast cell subpopulations, with specific roles in the process of placentation and in placental function. Cytotrophoblast cells (CTBs) are progenitor cells which constantly proliferate and differentiate into other trophoblast populations. By fusion, CTBs give rise of multinucleated syncytiotrophoblast (STB) and through epithelial-mesenchymal transition, CTBs differentiate to invasive extravillous trophoblast cells (EVTs). STB covers chorionic villi and is in direct contact with the maternal circulation, facilitating the exchange of nutrients, gases and excretory materials between the mother and fetus. EVTs invade the decidual stroma and part of the myometrium anchoring the placenta to the mother’s uterus while a portion of the EVTs, representing endovascular EVT cells (eEVTs), reaches the decidual spiral arteries, and transforms their walls by degrading and replacing the endothelial and smooth muscle cell layers. Inadequate implantation and impaired trophoblast differentiation and function lead to placental dysfunction and the development of different pregnancy complications which affect the maternal health and fetal development, with a possible lifelong impact on the offspring health (all in [1]).

The highly regulated and coordinated feto-maternal communication is essential for a successful pregnancy. Cytokines, small multifunctional molecules abundantly expressed at the feto-maternal interface, are one of the key players involved in this finely orchestrated interaction [2]. Data presented in the literature, together with our previous research, show that interleukins (IL)-6 and IL-8, among other cytokines, are one of the decisive drivers of the physiological pregnancy-related processes and pathological conditions [2].

IL-6 is a pleiotropic cytokine, a member of the IL-6 family of cytokines, implicated in a wide range of physiological processes, such as organ development, acute-phase response, inflammation, immune responses, metabolic regulation and others [3]. IL-6 exerts its effects upon binding to its receptors and subsequently activating the Janus kinase-signal transducer and activator of the transcription (JAK/STAT) pathway [4]. The activated STAT3 downstream induces the expression of the suppressor of the cytokine signaling 3 (SOCS3), a potent negative regulator of the JAK/STAT signaling that prevents excessive inflammation [5]. The classical signal transduction of IL-6 is induced by the binding of IL-6 to its specific membrane IL-6α-receptor (IL-6R), which is followed by the formation of a signaling complex with the signal-transducing receptor β-subunit, transmembrane glycoprotein 130 (gp130). This pathway is thought to be limited to a few tissues only, due to the restricted expression of IL-6R [6]. The signaling receptor β-subunit is shared by several other cytokines that comprise the IL-6 cytokine family, enabling redundant effects upon engaging with different ligands [6]. By contrast, IL-6 trans-signaling denotes the IL-6-binding to a soluble form of IL-6Rα (sIL-6R), which enables increased IL-6 bioavailability and broadening of its target cell repertoire, due to the ubiquitous expression of gp130 [6]. IL-6 and other members of this cytokine family may exert both pro- and anti-inflammatory functions. It is generally accepted that the pro-inflammatory effects of IL-6 are exerted by IL-6 trans-signaling, via the sIL-6R [6]. Moreover, the anti-inflammatory effects of IL-6 are mainly conducted via the classic membrane-bound IL-6R-mediated signaling [6]. The IL-6 signaling pathways and functions are described in more detail elsewhere [6,7].

IL-8, also known as C-X-C motif chemokine ligand 8 (CXCL8), is a pro-inflammatory chemokine, a member of the CXC family of chemokines, produced under inflammatory conditions by immune and other cell types [8]. The most prominent role of IL-8 is the attraction of neutrophils to the sites of inflammation, but also the promotion of monocyte-macrophage growth and differentiation [9], endothelial cell survival, proliferation and angiogenesis [10]. IL-8 also enhances the oxidative metabolism and generation of reactive oxygen species, possibly leading to oxidative stress [11]. The biological effects of IL-8 are induced upon engaging with its transmembrane, G protein-coupled receptors CXCR1 and CXCR2 [12], and the activation of the inflammatory Akt/protein kinase B (PKB), mitogen-activated protein kinase (MAPK) and protein kinase C (PKC) pathways [13].

**Figure 1:**
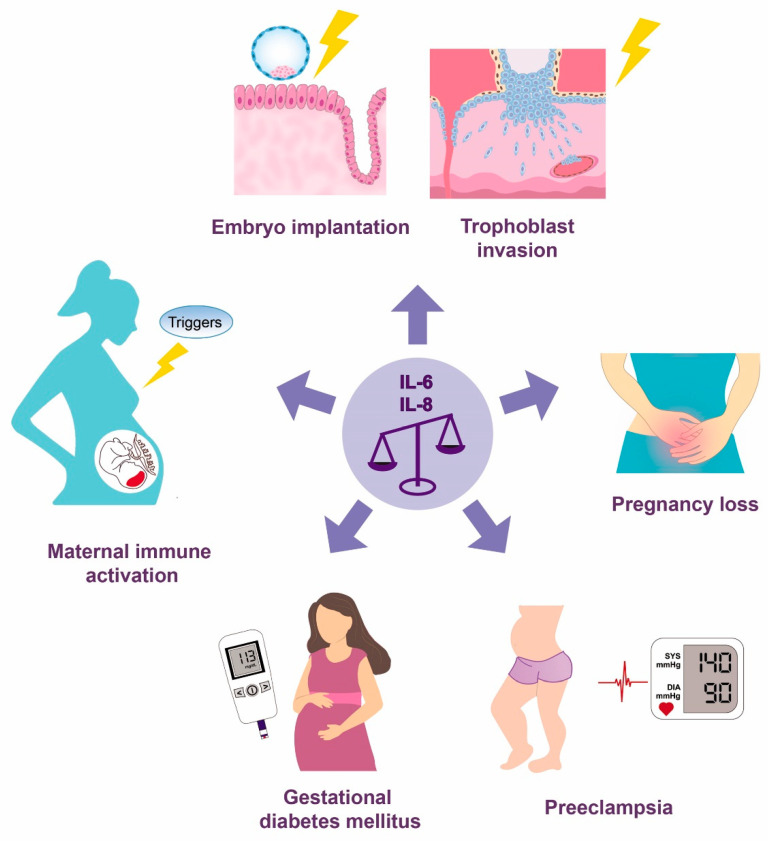
Implication of the IL-6 and IL-8 disbalance in the embryo implantation and trophoblast invasion, and the pathological pregnancy conditions – pregnancy loss, preeclampsia, gestational diabetes mellitus and maternal immune activation, based on the literature data.

This review discusses the role of IL-6 and IL-8 in processes important for the establishment of a healthy pregnancy and for parturition, as well as the involvement of these cytokines in the development of selected common pregnancy-related pathologies (Figure 1).

## 2. IL-6 and IL-8 in a Healthy Pregnancy

### 2.1. Expression of IL-6 and IL-8 and Their Receptors at the Feto-Maternal Interface

IL-6, IL-8 and their respective receptors are expressed in the human endometrium throughout the menstrual cycle [14,15,16]. IL-6 and IL-8 are predominantly localized to the endometrial epithelial and glandular cells [14,15]. IL-6R and gp130 are expressed in the endometrial glands [14], while CXCR1 and CXCR2 are immunolocalized into the surface of the endometrial epithelium, endometrial glandular cells, and, to a lesser extent, on stromal cells [16]. Both IL-6 and IL-8 show a menstrual cycle-dependent expression pattern, suggesting their role in endometrial physiology. IL-6 is weakly expressed during the proliferative phase, but its expression progressively increases after ovulation and peaks during the mid- to late-secretory phase [14]. This expression pattern temporally corresponds to the window of implantation, suggesting a role for IL-6 in the endometrial receptivity. Moreover, across the menstrual cycle, the IL-8 mRNA expression peaks at the early- to mid-proliferative phase and once again at the late secretory phase [15]. The first peak-expression suggests a role for IL-8 in the neovascularization of the growing endometrium, whereas the second peak could be related with the neutrophil recruitment into the endometrium, right before the onset of menstruation [15].

In the first trimester of pregnancy, IL-6 expression is detected in different cell populations from the uteroplacental tissues. In the decidua, decidual stromal cells (DSCs) and different populations of immune cells were immunostained for IL-6 [17,18,19]. In the placenta, IL-6 expression was found in CTBs, STB and EVTs [17,19,20]. In line with immunohistochemical analyses, IL-6 expression at the mRNA and protein level was also shown in isolated first-trimester CTBs and EVTs [20,21], as in primary DSCs, decidual natural killer cells (dNKs), CD8^+^ T cells and macrophages [18,20]. Moreover, IL-6 production by DSCs, CTBs and EVTs, is shown to increase with gestational age [20]. In first-trimester decidual sections and isolated cells, IL-8 is shown to be expressed in DSCs and the glandular epithelium, as well as in dNKs, decidual CD8^+^ T cells and macrophages [18,22,23,24]. In the first-trimester placenta, this protein is detected in different trophoblast subpopulations, as STB, CTBs and EVTs [22,24,25,26]. Study on explants from the first and the second trimesters, and the at term placenta, show that IL-8 secretion increases during gestation, with the maximal production in the second trimester and at term [25]. A widespread expression of IL-6R, gp130, CXCR1 and CXCR2 is found in first-trimester placental bed sections [18]. Among the trophoblast subpopulations, all of the named receptors were immunolocalized to EVTs [20,24].

### 2.2. Pregnancy Establishment

Considerable data now indicate that uterine epithelium-derived factors control embryo-implantation processes. In turn, the developing embryos have been shown to produce a variety of cytokines that may act in an autocrine fashion or on the endometrium to influence its receptivity [27]. The IL-6 immunoreactivity in the human endometrium is strong during the putative window of implantation, suggesting its role in this process [14]. A microarray analysis of the mid-secretory phase endometrium of patients suffering from recurrent implantation failure show a lower expression of IL-6, compared to healthy controls [28], further supporting the role of IL-6 in endometrial receptivity. Blastocysts also express and secrete IL-6 [29,30,31,32], and IL-6 in vitro treatment increases the embryos’ blastulation and hatching rates, compared to untreated embryos [33]. Furthermore, a higher IL-6 level in the follicular fluid also correlates with implantation success in patients undergoing in vitro fertilization (IVF) [34]. However, studies on animal models regarding the role of IL-6 in pregnancy establishment and maintenance are inconsistent. Reduced fertility, a decreased number of viable implantation sites and the increased rate of miscarriage in mid-gestation [35], but also no changes in fertility, implantation or early embryonic development [36], have both been observed in IL-6 knockout mice, compared to control mice. These discrepancies are suggested to reflect the differences in the lines of the IL-6 knockouts used in the studies, housing conditions, and other factors that influence the immune system development [36]. 

The results from our study and those by other groups also highlight IL-6 as one of the major regulators of multiple cellular processes at the feto-maternal interface. Namely, we have shown that IL-6 stimulates trophoblast invasion and migration of both primary first trimester CTBs and the EVT cell line HTR-8/SVneo [21]. This was partly mediated by the upregulated trophoblast expression of integrin α5, α1 and β1 [21], and the activation of MMP-2 and MMP-9 [37,38], molecules, particularly relevant for trophoblast invasion [39,40,41,42]. Our findings were also confirmed on other trophoblast cell models, such as ACH-3P and JEG3 cell lines [43,44]. However, Champion and collaborators did not observe a stimulatory effect of HTR-8/SVneocell invasion by IL-6 [20]. This discrepancy could be due to the different experimental conditions, compared to the ones found effective in our study [21].

Further, a role for IL-6 in the remodeling of spiral arteries has also been indicated. IL-6, along with IL-8, has been proposed as a key EVT-derived factor that activates endothelial cells to release chemoattractants for the dNKs and macrophages from the surrounding tissue into the upper segments of the spiral arteries, to initiate the remodeling process [45]. Along with mediating the trophoblast invasion and spiral artery remodeling, IL-6 has also been shown to mediate the immune-endocrine crosstalk in pregnancy. Namely, IL-6 was found to regulate the synthesis of the β-subunit of the human chorionic gonadotropin and human placental lactogen, two major placental hormones essential for pregnancy [46,47].

IL-8 is also proposed to contribute to endometrial receptivity and to participate in the dialogue between the embryo and the human endometrium during implantation. Namely, the endometrial IL-8 mRNA expression starts to increase at the receptive phase of the menstrual cycle [15,48]. Furthermore, in vitro findings demonstrate an upregulation of the IL-8 and CXCR1 expression in endometrial epithelial cells in the presence of an embryo [48,49]. Additionally, in vitro decidualization of endometrial stromal fibroblasts is shown to increase their IL-8 secretion in respect to the non-differentiated cells [50]. In patients undergoing IVF, IL-8 is suggested to be a predictor of the embryo developmental potential in the pre-transfer assessment of embryos. The pregnancy and the implantation rates, as well as the number of live births per IVF or intracytoplasmic sperm injection, were higher when the pre-transfer embryo-conditioned medium contained IL-8 [51]. 

Moreover, IL-8 is shown to stimulate progesterone secretion from the BeWo trophoblast cell line, suggesting a role for IL-8 in the maintenance of pregnancy [52]. dNKs are shown to regulate human trophoblast invasion in vitro as well as in in vivo mouse models by the production of IL-8 [23]. Recombinant IL-8 is shown to stimulate both first-trimester CTBs and HTR-8/SVneo-cell invasion [24,26], while silencing of IL-8 mRNA had the opposite effect [53]. The levels of the secreted MMP-2 and MMP-9 were significantly elevated by IL-8 treatment, as was the trophoblast expression of integrin α5 and β1, compared to the non-treated cells [24,26]. Furthermore, the viability and proliferation of HTR-8/SVneo cells increased following IL-8 treatment [26,54], as well as HTR-8/SVneo- and JEG-3-cell migration [26,55]. IL-8 has also been designated as one of the key EVT-secreted factors involved in spiral artery remodeling [45]. This chemokine is also shown to stimulate the endothelial-like phenotype in HTR-8/SVneo cells, reflected in an increased tube formation on Matrigel [54]. The presented literature data about the IL-6 and IL-8 roles in pregnancy establishment are summarized in Table 1.

In conclusion, the spatial and temporal distribution of IL-6, IL-8 and their corresponding receptors, clearly indicate a role for IL-6 and IL-8 signaling in the early events of pregnancy establishment—from the initial crosstalk between the embryo and the endometrium, to the subsequent formation of the functional placenta. Their disturbed expression in a range of clinical situations discussed further herein, suggests that manipulation with IL-6, IL-8, their receptors and/or components of their effector pathways, may be a plausible strategy for achieving a successful pregnancy in selected patients.

### 2.3. Parturition

Human labor is a physiological process that involves a coordinated activation and transformation of several reproductive tissues – myometrium, cervix and choriamniotic membranes. The timely development of the inflammatory/immune response in all main reproductive tissues involved in parturition, is shown to be indispensable for initiating the uterine contractions, cervical ripening and dilatation, as for the activation and rupture of the gestational membranes [77]. Recent concepts suggest that human parturition occurs when the upregulated pro-inflammatory mediators are amplified above a threshold level that stimulates the uterine transition to its activated state for labor [78,79]. This paracrine inflammatory amplification is based on multiple positive feedback loops between ligands, cells, and tissues [77]. Due to cellular stress, necrosis and senescence, more and more damage-associated molecular patterns (DAMPs) are released from the cells of the increasingly stressed uterus, maturing fetus and ageing placenta, as parturition nears [80]. Engaging with toll-like receptors (TLRs), DAMPs cause immune cell activation and inflammasome assembly, a release of pro-inflammatory cytokines and chemokines, and leukocyte chemoattraction and activation [81]. The activated leucocytes, along with the resident cells, jointly release inflammatory mediators, such as cytokines, chemoattractants, prostaglandins and other molecules. This enables the transition from a quiescent to an active uterine phenotype [78,79,82]. Consistently, a number of studies report increased mRNA expression and protein levels of IL-6 and IL-8 in the myometrium, cervix, decidua, amnion and choriodecidua, during labor [57,83,84,85,86,87]. This is supported by transcriptome studies showing upregulation of the relevant signaling pathways in the laboring gestational tissues [57,88,89,90,91,92].

According to the gene-gene association analysis, *IL6* might be the key gene to trigger specific mechanisms in the gestational tissues that eventually lead to the onset of labor [56]. This study showed that *IL6* was linked to genes which stimulate proliferation, maturation, chemoattraction and activation of neutrophils, including *CXCL8* [56]. Furthermore, IL-6 participates in the initiation and progression of parturition by stimulating the production of prostaglandins and oxytocin, which facilitate cervical ripening and induce uterine contractions. IL-6 is shown to stimulate prostaglandin synthesis by decidual cells and chorioamniotic membranes, the major sources of intrauterine prostaglandins [58,59]. Additionally, IL-6 treatment increases oxytocin secretion by myometrial smooth muscle cells (MSMCs) [60] and the expression of oxytocin receptors on them [61], thus establishing a positive feedback loop for amplifying the oxytocin-induced effects. Vice versa, oxytocin activates the master inflammatory transcription factor NFκB, followed by an upregulation of gene expression for IL-6, IL-8 and other inflammatory molecules in MSMCs [93], further amplifying the inflammatory process. In addition, the mechanical stretching of the uterus significantly elevates the expression of pro-inflammatory cytokines, such as IL-6 and IL-8 [94]. Nonetheless, according to rodent models, IL-6 alone is incapable of stimulating uterine contractions [95], or to induce preterm labor (PTL) [96].

An analysis of the inflammatory signaling pathways has shown that *CXCL8* is the most upregulated gene in both the myometrium and cervix with labor onset [57]. Consistently, the cells of the cervix and the lower segments of the uterus produce great amounts of IL-8 with the progression of the cervical dilatation and parturition [86,97,98]. In cervical stroma, expression of CXCR1 and CXCR2 was seen only after vaginal delivery [99], possibly providing a feedforward mechanism for amplification of IL-8 signaling during human vaginal parturition. Upregulation of IL-8 expression in the laboring intrauterine tissues is associated with an increased leukocyte infiltration [85,100]. In addition to neutrophil chemoattraction, IL-8 also stimulates neutrophil degranulation and release of MMPs and elastase, leading to extracellular matrix degradation [101,102]. This contributes to cervical remodeling and rupture of the gestational membranes—processes that promote spontaneous labor [62,63]. In line with that, a correlation between IL-8 expression and the neutrophil-derived MMPs in laboring tissues was detected [97,103]. 

Considering all the aforementioned (summarized in Table 1), it may be hypothesized that human parturition is driven by multiple local interactions between the pro-inflammatory and pro-contractile mechanisms. This perpetuates the inflammation towards a point-of-no-return value which sets in motion parturition cascades [77]. Although IL-6 and IL-8 are repeatedly implicated in various steps of labor, the exact role of each of them is still unclear. Further research on the specific inflammatory interactions associated with the onset and progression of parturition could lead to a better risk assessment and treatment of PTL. For instance, tracking IL-8 serum levels in pregnant women is suggested as a main marker to determine the time of parturition [104]. In addition, the maternal IL-6 and IL-8 serum levels are also considered as appropriate markers for monitoring the effects of tocolytics in PTL [105]. Being central to the initiation and propagation of the inflammatory signaling cascade in parturition, IL-6 and IL-8 may also serve as potent therapeutic targets for PTL and other adverse birth outcomes. As suggested, targeting the inflammatory cascade at an earlier stage, could be a promising tocolytic strategy [82,106].

### 2.4. Circulating IL-6 and IL-8 Levels in a Healthy Pregnancy

Measurements of the circulating cytokine levels show that IL-6 levels are generally found to be elevated in pregnant vs. non-pregnant women [67], especially in the second and third trimester [66,67]. Longitudinal assessments of IL-6 concentrations in the maternal circulation over the course of pregnancy, adjusted for maternal body mass index (BMI) and other confounders, yield rather conflicting findings (Table 1). While several studies report a progressive increase of IL-6 with advancing gestational age [64,65,66,67,69], no significant differences between trimesters [68,70,71,72], or a decrease in the circulating IL-6 levels during gestation [73,74], have also been observed.

The maternal serum levels of IL-8 have been shown to decrease with gestational age during the first half of a non-complicated pregnancy [69,76], but to increase between the second and third trimester [68,70] (Table 1). This pattern of circulatory IL-8 levels might suggest a Th1/Th2 cytokine shift towards a pro-inflammatory profile, as the term for parturition approaches, following the predominantly immunotolerant state that protects the feto-placental unit from the maternal immune system [107]. Still, a progressive decline in serum IL-8 over the course of pregnancy has also been observed [75].

Although the influence of the fetoplacental sex on the maternal immune milieu is growingly recognized [108,109,110], only a few existing studies investigated gestational cytokine levels in respect to the fetoplacental sex. Although data indicate a generally more pro-inflammatory milieu in women carrying male vs. female fetuses [108,110], no significant sex-specific differences regarding the IL-6/IL-8 levels under steady state conditions are reported [108,109,110,111]. However, upon lipopolysaccharide (LPS) stimulation, a more robust inflammatory response, reflected in greater production of cytokines, including IL-6, was shown in cultures of peripheral blood mononuclear cells (PBMCs) from mothers carrying female vs. male fetuses, at all tested time-points across pregnancies [109]. In summary, it may be concluded that the circulating IL-6 and IL-8 levels fluctuate over the course of pregnancy. This may reflect the continuous immune modulation across gestation, consistent with the aim of pregnancy maintenance. Identifying typical patterns in immune parameter trajectories over the course of pregnancy should enable recognizing relevant deviations and predict adverse perinatal outcomes [69]. However, comparisons of absolute cytokine levels between studies are often inconclusive in practice. The abundance of inconsistent data probably reflects the methodological inconsistencies between the studies and inter-assay differences (i.e., the detection of varying amounts of free and/or bound cytokines, using plasma vs. serum, differences in population size—statistical power, population characteristics), and other factors which cannot be corrected [67,70]. Conflicting results may also be related to a number of factors that influence cytokine levels, such as age, ethnicity, genetic polymorphisms and epigenetic marks, fetoplacental sex, pre-gestational BMI, HbA1c, diet, smoking, intestinal microbiota, and other [112,113,114,115]. Therefore, more standardized fundamental mechanistic research and longitudinal study designs accounting for confounding variables may help to clarify the role of IL-6/IL-8 in a healthy pregnancy and related disorders. Nevertheless, a panel of cytokines/chemokines and other related parameters, adjusted for the multicollinearity among them, is more likely to describe the inflammatory milieu in pregnancy and predict the outcomes than the individual measures.

## 3. IL-6 and IL-8 in Selected Pregnancy Pathologies

### 3.1. Pregnancy Loss 

Pregnancy loss (PL) is the most common pregnancy complication. The pooled risk of PL is 15.3% of all clinically recognized pregnancies, usually before the 12th week of gestation (wg) [116]. Most PLs stay undetected since they happen soon after implantation. With these cases included, the incidence of PLs rises to 30% [117,118]. Spontaneous PLs are usually sporadic (SPL), but 1% to 5% of women experience recurrent PLs (RPL). RPL is defined as more than two or three consecutive PLs, depending on the definition [119]. Genetic abnormalities of the conceptus represent a major cause of early PL [119]. Other common risk factors and causes of PL, especially recurrent, include maternal anatomical malformations, infections, endocrine, thrombophilic and immune disorders [119]. Nevertheless, the etiology of more than 50% of PLs remains unexplained [120].

An inadequate expression/secretion of IL-6 and IL-8 at the feto-maternal interface has been indicated in unexplained early PLs in several studies (Table 2). In isolated SPL, decidual macrophages and dNKs are found to produce less IL-6 and IL-8, compared to the corresponding cells from a normal pregnancy [18]. Considering that: (i) IL-6 and IL-8 are involved in the regulation of trophoblast invasion and spiral artery remodeling [18,21,26], and (ii) dNKs stimulate EVT invasion, at least partly, through IL-8 signaling [24], suboptimal decidual IL-6 and IL-8 levels could lead to an inadequate trophoblast invasion and spiral artery remodeling, and eventually to an early PL. However, recent studies report increased IL-6 and IL-8 expression in decidual tissue [121,122,123], and increased IL-8 expression in decidual macrophages and dNKs [124,125], in RPL, compared to normal pregnancy. Furthermore, increased IL-8 levels in products of conception containing tissue of maternal and fetal origin, were reported in RPL patients, compared to healthy controls [126]. Increased IL-6 and IL-8 levels in decidual tissue indicate an enhanced pro-inflammatory state at the feto-maternal interface that could be detrimental to the implanted embryo and compromise the pregnancy. The previous findings indicate that both insufficient and excessive levels of IL-6/IL-8 disturb the inflammatory network at the feto-maternal interface, which may compromise the pregnancy. Furthermore, the differences in the expression profile of IL-6 and IL-8 in reproductive tissues between SPL and RPL, support the hypothesis that these complications may have a substantially different etiopathogenetic background [127].

Patients experiencing PL may exhibit altered systemic cytokine levels, compared to women having uneventful pregnancies, although the results are varying significantly. An increased IL-6 concentration in plasma [128] and serum [123,129,130], as well as greater expression in PBMCs [123,128], is found in SPL and RPL patients vs. controls. Unaltered [131,132] or decreased IL-6 levels in serum [133,134,135,136] of SPL and RPL patients are also shown. Similarly, unaltered [131], increased [133,136] and decreased [134] levels of IL-8 in PL patients are all reported. These inconsistent findings could be explained, at least in part, by the methodological differences between the studies (inclusion criteria for the participants, gestational age, inclusion of both SPL and RPL patients in the study, assigned control groups, etc.).

Women experiencing RPL exhibit a heightened immune activity, both locally and systemically, regardless of the gestational status. Thus, in non-pregnant RPL patients, higher IL-6 plasma levels, compared with women without reproductive problems [137], along with increased circulating levels of sIL-6R [138] are found. This, in addition to the lower levels of soluble gp130 (sgp 130) *—* a selective antagonist of the IL-6/sIL-R trans-signaling pathway [139], points to increased pro-inflammatory IL-6 trans-signaling in RPL patients [138]. Moreover, the PBMCs of RPL patients isolated at the mid-luteal phase of the cycle, which corresponds to the window of implantation, were shown to more readily respond to stimulation, expressing a greater amount of IL-6 mRNA, compared to healthy controls [140]. Increased IL-8 mRNA and protein levels in peripheral blood samples of non-pregnant RPL patients vs. controls are also reported [141,142].

Locally, the peri-implantation endometrial tissue of RPL-prone patients is shown to express lower IL-6 and IL-8 levels, compared to controls [143,144,145,146]. The impaired expression of IL-6, IL-8 and other cytokines in the mid-secretory endometrium could affect endometrial receptivity and thus compromise the establishment of a pregnancy. Or, it may impair the decidual selection of high-quality embryos, which could lead to a subsequent abortion.

Taken together, the reported data suggest that IL-6 and IL-8 may be closely associated with PL, possibly through different underlying mechanisms, consistent with the multifactorial nature of PL (Table 2). The variety of findings regarding the local or systemic IL-6 and IL-8 expression between PL-prone women and controls, suggests that neither IL-6 nor IL-8 could be a sole underlying factor of PL. Hence, neither IL-6 nor IL-8 levels alone represent a reliable diagnostic test for PL. Instead, a panel of several cytokines and/or other parameters in combination may provide a greater predictive value than any single factor utilized alone. Furthermore, a better understanding of the specific underlying mechanisms of PL is also needed, to explore the therapeutic potential of the IL-6 and IL-8 pathway regulation in PL-prone patients. The previous implies a more personalized approach towards the diagnostics and the therapeutic treatments—ideally, adjusted to the individual patient.

**Table 2:** Pathology-related changes of the IL-6/IL-8 in pregnancy pathologies.

Pathology	Sample	Pathology-Related change	Cytokine	Reference
**Pregnancy loss**	Decidual macrophages and dNKs *	Decreased expression in SPL *	**IL-6, IL-8**	[18]
		Increased expression in RPL *	**IL-8**	[124,125]
	Decidua	Increased expression in RPL *	**IL-6, IL-8**	[121,122,123]
	Serum	Increased concentration	**IL-6**	[123,129,130]
			**IL-8**	[133,136]
		No change	**IL-6**	[131,132]
			**IL-8**	[131]
		Decreased concentration	**IL-6**	[133,134,135,136]
			**IL-8**	[134]
**Preeclampsia**	Placenta	Increased expression	**IL-6**	[147,148,149,150,151]
			**IL-8**	[149,152,153]
	Serum	Increased concentration	**IL-6**	[150,151,154,155,156,157,158]
			**IL-8**	[151,153,156,159,160,161,162]
**Gestational diabetes mellitus**	Placenta	Increased expression	**IL-6**	[115,163,164,165,166]
			**IL-8**	[115,167]
		No change	**IL-6**	[168,169]
			**IL-8**	[169,170]
		Increased levels in extravillous and decreased in villi tissue	**IL-6**	[167]
		Sex-specific expression in STB * and EVTs *	**IL-8**	[171]
	Serum	Increased concentration	**IL-6**	[113,115,172,173,174,175,176,177,178,179]
			**IL-8**	[115,176,179,180]
		No change	**IL-6**	[180,181,182,183]
			**IL-8**	[167,184,185]
		Decreased concentration in early pregnancy	**IL-8**	[184]
**Maternal immune activation**	Placenta/trophoblast and fetal membranes	Increased expression in response to inflammatory stimuli	**IL-6**	[186,187,188,189,190,191]
			**IL-8**	[189,190,191]
	Amniotic fluid and cervicovaginal lavage	Increased concentration in API *	**IL-6**	[192,193,194,195,196,197,198]
			**IL-8**	[192,193,194,195,196,197,199,200]

* API—acute placental inflammation; dNKs—decidual natural killer cells; EVTs—extravillous trophoblast cells; RPL—recurrent pregnancy loss; SPL—sporadic pregnancy loss; STB—syncytiotrophoblast.

### 3.2. Preeclampsia 

According to the 2018 recommendations from The International Society for the Study of Hypertension in Pregnancy (ISSHP), preeclampsia (PE) is defined as de novo hypertension after the 20th wg, accompanied by one or more of the following features: proteinuria, maternal organ dysfunction (including hepatic, renal, neurological), or hematological involvement, such as thrombocytopenia, and/or uteroplacental dysfunction, such as fetal growth restriction (FGR) and/or abnormal Doppler ultrasound findings of the uteroplacental blood flow [201]. PE affects approx. 2% to 8% of pregnancies worldwide [202]. Maternal and perinatal outcomes in PE are predicted based on the gestational age at the onset. Accordingly, PE is classified as early-onset PE (EOPE), occurring before the 34th wg, and late-onset PE (LOPE), which manifests at or after the 34th wg [201]. LOPE comprises around 80% to 95% of all PE cases, while EOPE, although less common, is associated with a higher maternal morbidity and FGR or neonatal mortality rates [203,204]. Although the exact mechanisms are not fully understood, there are indications that EOPE is related to abnormal placental development and consequent placental inflammation/dysfunction, whereas LOPE seems to be related to inherent maternal cardiovascular dysfunction and systemic inflammation [204,205].

Common features of the both PE phenotypes are placental ischemia and increased oxidative stress, with excessive systemic inflammation and endothelial dysfunction, which may be deleterious to the fetal and maternal health [206]. A reduced trophoblast invasion into the decidua and defective spiral artery remodeling are thought to be the earliest pathophysiological events in PE [207]. The shallow trophoblast invasion and development of placental hypoxia, induced tissue injury and increased release of inflammatory mediators from the placental cells [207]. The increased levels of pro-inflammatory cytokines, reactive oxygen and nitrogen species, lytic enzymes and other aggressive molecules damage the endothelial cells, causing their dysfunction and increased endothelial production of vasoconstrictors over vasodilators, leading to maternal hypertension and uteroplacental dysfunction [208]. An analysis of the dynamic connections within the pro-inflammatory cytokine network in PE cases identified a positive correlation between IL-6 and IL-8, suggesting these cytokines are implicated in the pathophysiology of PE [156] (Table 2). Consistent with the previous notion, IL-6 is recognized as a circulating marker of endothelial dysfunction and increased levels have been observed in the sera of women suffering from PE [150,151,154,155,156,157,158]. Interestingly, besides IL-6, increased level of sgp130 in the maternal circulation [209] and lower release of sIL-6R from the maternal neutrophils [210] are also shown in PE patients, compared to healthy pregnant women. As previously suggested, these findings possibly indicate a compensatory mechanism to control IL-6 signaling and prevent an overactivation of the IL-6/sIL-6R pathway [211,212].

An increased IL-6 expression is found in decidual cells and placentas of PE patients, associated with elevated levels of plasma IL-6 in PE [147,148,149,150,151]. The local excess of IL-6 could increase the trophoblast shedding, as shown in vitro, possibly contributing to the development of PE [213]. Furthermore, IL-6 aids to the recruitment and activation of the decidual macrophages that could lead to disturbed EVT invasion and spiral artery transformation [147,214,215]. A shift in the macrophage differentiation from the anti-inflammatory M2 to the pro-inflammatory M1 phenotype is observed in PE deciduas [151,214,216], consistent with a greater production of pro-inflammatory cytokines and decreased levels of anti-inflammatory cytokines in PE placentas [150,151,214,216]. In addition, an excess of IL-6 favors the differentiation of naïve CD4^+^ T cells towards Th17 and cytotoxic T cell phenotype, whereas it inhibits the differentiation of Th2 and regulatory T (Treg) cells [217,218]. This contributes to the immune maladaptation and sustained systemic inflammation observed in PE [219]. Therefore, applying IL-6-reduction strategies as anti-IL-6 monoclonal Abs (mAbs) or TLR inhibitors to the treatment of PE, could shift the differentiation of naïve CD4^+^ T cells towards the anti-inflammatory Treg and Th2 phenotype, rather than the pro-inflammatory Th1 and Th17 one [219]. Of note, although the current data do not indicate a substantially increased malformation risk of using anti-IL-6 mAbs in pregnancy, they are insufficient to prove safety [220,221].

IL-8 is considered to contribute to the PE pathogenesis by attracting more neutrophils into the endothelium [222]. Neutrophils infiltrate the vessel tunica intima and release reactive oxygen species, myeloperoxidase, MMP-8 and thromboxane, causing cell injury/death, endothelial inflammation and vasoconstriction [156]. Neutrophil extracellular traps (NET) have been found in placental intervillous spaces in PE patients [223]. The presence of NETs in the maternal circulation during pregnancy can contribute to thrombotic events, inflammation, and ultimately, to fetal death [224]. Consistently, higher IL-8 serum levels [151,153,156,159,160,161,162], and placental tissue expression of IL-8 [149,152,153] are reported in PE patients, compared to healthy controls.

According to some studies, sex-specific susceptibility is noticed for different pregnancy complications, including PE [225]. Hence, pregnant non-Asian women bearing male fetuses were found to have an increased risk for developing PE [226]. Moreover, the placental inflammatory response in PE was found to be significantly influenced by the fetal sex [149]. For instance, the IL-6 and IL-8 expression was found to be more pronounced in male, compared to female PE placentas [149]. Of note, healthy placentas did not exhibit any sexual dimorphism in the expression of IL-6/IL-8 [149]. The underlying mechanisms of the reported sexual dimorphism in PE remain unclear, but they could be associated with sex-specific gene expression in early to mid-gestation placenta [227]. In that vein, placental transcriptome profiling revealed that genes upregulated in male placentas are the ones involved in the regulation of the immune response [227]. 

Conflicting results are also shown for the serum levels of IL-6 and IL-8, when comparing severe vs. mild PE, or the time of PE onset. A number of studies reported elevated levels of both cytokines in severe, compared to mild PE [162,228,229,230,231,232]. However, an absence of an association between the maternal serum IL-6 levels and the severity of PE is also reported [155,233,234]. Comparing maternal blood concentrations of IL-6 between EOPE and LOPE on the one hand, and a healthy pregnancy on the other, also yielded inconsistent findings [205,232,235,236]. These inconsistencies could reflect individual differences in age, hormonal status, lipid concentration, chronic inflammation and other factors which are shown to affect individual circulating IL-6 levels [237,238,239], and whether these confounders were factored into the analyses or not. 

Collectively, it can be concluded that the altered trophoblast invasion and spiral artery remodeling, as well as the endothelial dysfunction in PE, are interrelated with the immune maladaptation and disturbed homeostasis of IL-6 and IL-8. However, what remains unresolved is to what extent the levels of these cytokines relate to the severity of PE and its phenotypes, due to a large number of studies with conflicting results. The latter probably reflects not only the inconsistencies between the study methods, but also the multifaceted nature of the PE syndrome and the heterogeneity of risk factors and mechanisms leading to its development. In that context, PE, or at least EOPE, is seen as just one in a spectrum of complications of pregnancy that share a common pathophysiology rooted in aberrant placentation. In general, an elevated level of pro-inflammatory cytokines in the maternal circulation, with a shift in the “IL-8 × IL-6” axis towards the pro-inflammatory Th1 response is thought to drive the cytokine network in PE women towards an excessive systemic inflammatory state [156]. Thus, while the mechanistic relevance of IL-6 and IL-8 in the pathogenesis of PE is to some extent obvious, the mechanisms influencing their dysregulation are noteworthy objectives of additional investigations.

### 3.3. Gestational Diabetes Mellitus 

Gestational diabetes mellitus (GDM) is a common gestational complication, discernable by de novo spontaneous hyperglycemia that develops during the course of pregnancy [240]. It is formally defined as “diabetes first diagnosed in the second or third trimester of pregnancy that is not clearly either preexisting type 1 or type 2 diabetes mellitus” (DM) [240]. GDM usually resolves following delivery, however, it may have long-lasting health consequences for the mother and the fetus, including an increased risk for type 2 DM and cardiovascular diseases [241,242]. It may be associated with premature delivery and PE [243,244], and quite often with neonate hypoglycemia, macrosomia and obstructed labor, due to the endogenous production of fetal insulin and insulin-like growth factor 1, in response to maternal metabolic alterations [245].

Analyses of the risk factors for GDM, despite methodological inconsistencies, point to an advanced maternal age, overweight/obesity, excessive gestational weight gain, ethnicity, genetic polymorphisms, low or high birth weight, family or past history of GDM, and other insulin-resistant states, such as polycystic ovarian syndrome, as common risk factors for developing GDM [246,247,248]. As in type 2 DM, insulin resistance (IR) and β-cell dysfunction play a central role in the pathophysiology of GDM [249]. In normal pregnancy, during early gestation, insulin sensitivity increases, promoting adipose storage of glucose in preparation for the increased energy demands in gestation [250]. As pregnancy advances, the surge of adipokines and diabetogenic placental hormones (progesterone, cortisol, prolactin and human placental lactogen) promotes a state of decreased insulin sensitivity and hyperglycemia [251]. Thus, a physiological pregnancy is considered an insulin-resistant state, with a 50% reduction in the insulin-mediated glucose clearance, and a more than two-fold increase in insulin production to maintain maternal euglycemia [252]. Chronic hyperglycemia and hyperinsulinemia, along with increased inflammatory and oxidative stress, is detrimental for the maternal pancreatic β-cells, leading to their functional exhaustion and injury/death [253]. When β-cell function fails to compensate the additional metabolic stress imposed by the diabetogenic state of pregnancy, the glucose metabolism becomes further dysregulated, leading to the development of GDM [254].

Evidence points that GDM is associated with changes in the maternal, fetal and placental inflammatory profile [254]. Systemic, chronic, subclinical inflammation that involves unbalanced cytokine production, is a key feature of GDM. Such metabolically induced inflammation, appropriately termed “metainflammation” [255], also accompanies obesity, IR, metabolic syndrome, type 2 DM and other related metabolic disorders. Although a lack of a significant association between the circulating IL-6 levels and GDM has been observed in some studies [180,181,182,183,256], an elevated concentration of IL-6 has been frequently reported in GDM patients, even regardless of obesity [113,115,172,173,174,175,176,177,178,179]. The results of the most recent systematic review indicate that serum IL-6 levels seem to be significantly higher in the majority of GDM patients, compared to euglycemic pregnant women [257]. Thus, as the authors suggest, assessing the serum IL-6 level could be a feasible diagnostic criterion for GDM [257]. As observed in PE and other chronic low grade inflammations [258,259], the increase in the systemic IL-6 in GDM patients may be accompanied by an increase in the sgp130 concentration [211]. This finding could be indicative of a compensatory anti-inflammatory mechanism to prevent overt inflammation induced by IL-6 trans-signaling [211,212]. Moreover, animal studies reveal that the blockade of peripheral IL-6 trans-signaling by recombinant sgp130, induces mature-onset obesity, glucose intolerance and IR [260]. 

The first (and, so far, the only) meta-analysis and systematic review of chemokines and their cognate receptors, suggests a role for IL-8 in the shaping of the complex immune microenvironment in GDM [261]. This is supported by case-control studies reporting increased circulating IL-8 levels in GDM patients, compared to healthy pregnancies [115,176,179,180]. However, comparable concentrations of IL-8 in the plasma of GDM patients vs. healthy pregnant women are also reported [167,184,185], as well as a lower IL-8 level in early pregnancy [184]. These inter-study inconsistencies probably emerge from the differences in the applied protocols and assays.

Current data point that both innate and adaptive immune system components respond to hyperglycemic and IR conditions, participating in the development of metainflammation [262]. In an obese state, the immune cells maintaining an anti-inflammatory environment in the adipose tissue are replaced with a pro-inflammatory immune-cell infiltrate [262]. This is accompanied by an increased secretion of pro-inflammatory cytokines and chemokines that act in an autocrine, paracrine, and endocrine manner, to promote inflammation and IR in the adipose and other target tissues [262]. Additionally, evidence shows that activated adipocytes are one of the main sources of the IL-6 and IL-8 production in obesity-associated IR and also in GDM [263,264]. In line with this, Kleiblova and coauthors indicated an upregulated IL-6 mRNA expression in subcutaneous adipose tissue of pregnant women with GDM [169]. Unlike TNF-α, which is suggested to act locally (in the adipose tissue) in an autocrine/paracrine manner contributing to the local IR and inducing IL-6 secretion, IL-6 rather appears to be released systemically by the adipose tissue, acting more as an endocrine signal that induces the hepatic acute-phase response and IR [265]. In fact, under basal conditions, up to 35% of systemic IL-6 is shown to originate from visceral adipose tissue in obese states, secreted by adipocytes and resident/infiltrated immune cells [265]. IL-6 contributes to IR primarily by impairing the phosphorylation of the insulin receptor and insulin receptor substrate-1, and inducing the expression of SOCS3–which impairs insulin signaling [266]. IL-6 is also known to promote lipolysis and secretion of free fatty acids from the adipose tissue into the circulation, which contributes to IR and to the increased gluconeogenesis in hepatocytes [267]. 

In obesity-related IR, visceral white adipose tissue (WAT) is considered to be the main source of IL-8, along with subcutaneous WAT and the infiltrated macrophages [268]. Considering that IL-8 attracts not only neutrophils and other immune cells, but also adipocytes, IL-8 secreted from hypertrophic adipocytes may contribute to the further accumulation of excess intra-abdominal fat in obesity [112]. Moreover, IL-8 itself enhances IL-8 mRNA expression in human adipocytes, thus providing an autoamplifying loop via CXCR1 and CXCR2 expressed on the human adipocytes [269]. Data suggest that persistent inflammatory stimuli may perpetuate this vicious circle of IL-8 production in human adipocytes over the p38 MAPK pathway, which is also implicated in promoting IR in human adipocytes [269]. Moreover, IL-8 in obesity may downregulate adiponectin in adipocytes [169,261]. A decreased adiponectin level is a common finding in IR, DM and GDM [270]. Given that adiponectin stimulates insulin secretion, enhances its signaling and inhibits gluconeogenesis [271], by decreasing the adiponectin production, IL-8 may play a crucial role in obesity-linked IR and GDM. Consistently, increased levels of IL-8 are detected in visceral adipose tissue of women with GDM [169,272,273].

The placental common repertoire of cytokines also becomes overexpressed in a diabetic environment [274,275,276]. Data from the literature show an increased IL-6 mRNA expression in GDM placentas [115,163,164,165,166], possibly associated with the enhanced macrophage infiltration in GDM placentas, compared to a physiological pregnancy [163,168]. However, no significant differences in the IL-6 mRNA expression in placentas of women with GDM, compared with healthy controls were also reported [168,169]. Results on the IL-8 placental expression in GDM patients are also conflicting. No significant difference [169,170], as well as increased IL-8 levels in GDM placentas [115,167], compared to healthy controls were both detected. Interestingly, one recent study revealed that the expression of IL-8 in GDM placentas might be sex-specific [171]. Namely, the male GDM placentas exhibited a lower IL-8 expression in EVTs and STB, compared to sex-matched controls [171]. On the other hand, the female GDM placentas expressed comparable and higher levels of IL-8 in EVTs and STB, respectively, than the sex-matched controls [171]. However, the implications of this sex-specific expression of IL-8 in GDM placentas remain to be elucidated. Data regarding the IL-6 and IL-8 expression/circulating level changes in GDM are summarized in Table 2.

Collectively, it may be concluded that a certain level of metabolically induced inflammation, reflected in the more pronounced maternal cellular and biochemical inflammatory profile than in non-diabetic pregnancies, accompanies maternal IR and GDM. IL-6 appears to be decisively involved in the development of IR and GDM, primarily by the impaired phosphorylation of the insulin receptors and the induced expression of SOCS3, which inhibits the insulin signaling in peripheral tissues, adipocytes and hepatocytes [266]. Moreover, IL-8 seems to participate in promoting obesity, inflammation and IR, by attracting additional adipocytes and immune cells in the adipose tissue, and by interfering with insulin signaling by downregulation of adiponectin and/or activation of the p38 MAPK pathway [112,261,269]. However, studies investigating inflammatory mediators in the maternal and placental compartments in GDM are not always consistent, whereas data regarding the fetal inflammatory state in GDM are lacking. A better understanding of the inflammatory process in GDM is urgently needed, considering that the placental inflammation in GDM may have a central role in shaping the in utero environment that “programs” the offspring development [169,277]. As the prevalence of pre-gestational DM and GDM continues to rise worldwide [278], such an understanding will be critical to optimize long-term health outcomes for both the mother and the offspring. In the meantime, it is necessary to once again stress the importance of correcting the modifiable risk factors and applying inflammation-reducing life-style changes during pregnancy, especially in the early stages of pregnancy, to reduce the risk of developing GDM and the consequences associated with it [279].

### 3.4. Maternal Immune Activation 

Maternal immune activation (MIA) in pregnancy is usually associated with acute infections, or with a sterile, low-grade, persistent inflammation, accompanying a number of systemic conditions, such as metabolic syndrome, type 2 DM, autoimmune diseases, cardiovascular disease, anxiety, depression, socio-economic adversity, micronutrient deficiencies, microbiome alterations, exposure to cigarette smoke or Δ-9-tetrahydrocannabinol, air pollution and other factors [280,281,282,283,284,285,286,287]. This myriad of exogenous and endogenous environmental exposures during pregnancy may cause tissue injury and trigger maternal inflammatory/immune responses, leading to a release of a plethora of effector molecules, with IL-6 and IL-8 having one of the key roles [288].

Maternal systemic infections in pregnancy are recognized as the principal non-genetic risk for neurodevelopmental and neuropsychiatric disorders in the child [289,290,291]. The variety of infectious agents associated with a shared neurodevelopmental risk points to the maternal immune response rather than a particular pathogen as a common denominator of the dysregulated offspring development [292,293]. This concept is particularly plausible considering that an increased risk of developmental abnormalities was also associated with pathogens that typically do not cross the placental barrier (influenza, agents causing upper respiratory infections) [280,294,295,296] or with serologic evidence of the maternal pre-gestational exposure to pathogens in the absence of an active infection [297,298,299]. It has been suggested that MIA during the sensitive window of in utero brain development, may alter neurodevelopmental trajectories, following the proposed sequence of events: (i) infection or other noxious stimuli trigger maternal inflammatory/immune responses, (ii) released cytokines and other inflammatory mediators cross the blood-placental barrier and (iii) activate the fetal immune system enabling the establishment of a self-propagating, low-grade inflammatory cascade [300]. Inflammatory cytokines reaching the fetal brain may trigger microglial activation and upregulation of pro-inflammatory transcription factors. Consequently, aberrant gene and protein expression may in long term give rise to neurological, immunological and behavioral disturbances in a predisposed offspring [300]. Supported by a fair amount of epidemiological data and animal research, IL-6 has been identified as the critical mediator in this unfortunate series of events [301,302,303,304].

Apart from systemic infection, acute placental inflammation (API), the microscopic equivalent to the clinical diagnosis of chorioamnionitis [305], is another common MIA-associated event in pregnancy, even in a clinically inapparent disease [306,307]. Low-stage API is observed in up to 50% of uncomplicated vaginal deliveries following uncomplicated pregnancies [308]. It is even more frequent in the absence of infectious agents, than due to intra-amnionic infection [192]. Whether the microbial invasion of the chorioamniotic membranes or the release of DAMPs during the course of cellular injury/death, an increase in the expression of IL-6 and IL-8 follows, along with the release of other acute phase mediators [25,193,309]. This is supported by findings of an increased expression of IL-6 and IL-8 in the trophoblast in response to LPS stimulation [188,190,191]. Consistently, elevated concentrations of IL-6 and IL-8 in amniotic fluid (AF) or cervicovaginal lavage, as indicators of API in PTL, are reported in a vast number of studies [192,193,194,195,196,197,198]. Additionally, a high concentration of IL-6 in AF was significantly associated with an increased risk of perinatal morbidity and mortality [197]. The AF level of IL-8 sampled in the third trimester of pregnancy was shown to correlate with the severity of API, irrespective of the presence/severity of funisitis [200], suggesting that the high AF IL-8 levels in API are mainly a product of the maternal immune response. Moreover, some studies indicate that human IL-8 does not seem to cross placentas obtained from pregnancies at term, implying that IL-8 in AF and in fetal blood is solely of fetal origin [310,311]. This is consistent with the data showing human fetal amnion and chorion as an important source of IL-8 in the setting of an ongoing inflammation [312,313,314]. The source of IL-6 in MIA has also been debated. Through a rodent MIA model, Hsiao and Patterson proposed that elevated level of IL-6 in the placenta was of maternal origin only, including both circulating IL-6 and the one secreted by the placental resident cells [189]. Nevertheless, the fetus itself can also mount an inflammatory response to maternal infection, especially in terms of IL-6 production [315,316]. Fetuses with fetal inflammatory response syndrome (FIRS), characterized by high levels of IL-6 in fetal plasma, had a higher rate of severe neonatal morbidity and a shorter procedure-to-delivery interval [317]. Considering these risks, rapid tests for the timely identification of inflammatory processes in the amniotic cavity that would circumvent amniocentesis as an invasive technique are highly needed in clinical practice. To this end, the determination of IL-8 [318] in the maternal serum and IL-8 [319] or IL-6 [318] in the cervical secretion for non-invasive screening for chorioamnionitis was suggested. However, there are data indicating that high levels of cervical IL-6 and IL-8 are only moderately predictive of intrauterine infection/inflammation and preterm delivery [194]. The reported data are summarized in Table 2.

The role of IL-6 and IL-8 in the pathogenesis of the neurodevelopmental and neuropsychiatric endophenotypes as MIA sequelae is widely supported by epidemiological studies indicating that in utero exposure to elevated concentrations of both, IL-6 [320,321,322], and IL-8 [323,324,325,326], may partially account for an increased risk of neurodevelopmental/neuropsychiatric disorders. However, remarkably little is known about the mechanistic pathways that connect these molecules with neurodevelopmental disorders. Emerging data suggest that both IL-6 and IL-8 inhibit synaptic long-term potentiation and induce changes in the hippocampal-dependent learning and memory tasks [327]. IL-6 may also influence the fate switching and cell differentiation in development, acting directly on the progenitor cells to regulate fetal neurogenesis and gliogenesis [328,329], or altering many parameters that influence neuronal migration, axonal pathfinding and synapse formation [330], or fetal growth in general, including nutrient transfer, anoxia and vascular permeability at the feto-maternal interface [331,332,333]. IL-8 dysregulation has also been found to have a role in atypical white matter development in preterm infants [334] and brain dysmaturation [326]. In addition, the activation of the JAK/STAT3 signaling axis by the maternal IL-6 in murine placenta, indirectly influenced fetal neurodevelopment through the diminished production of both the placental growth hormone and the insulin-growth factor 1 [189] indispensable for the proper fetal development [335]. Moreover, IL-6 can disrupt the immunological homeostasis of the placenta and the maintenance of the maternal tolerance by altering the Th1/Th2 ratio and by activation of the uterine immune cells [329,330]. 

One murine study employing a trophoblast IL-6R knockout model, reported no sexual dimorphism in the MIA-related behavioral abnormalities [303]. Noteworthy, it is generally recognized that MIA affects the offspring neurodevelopment in a sexually dimorphic manner and those sex-specific effects persists across the lifespan [320,336]. Sex differences in the placental responses to MIA, fetal brain structure/function and immune response could account for these sexually dimorphic effects of MIA [337,338,339]. Both human [322,340,341] and animal [336,342,343] studies indicate that male offsprings are more frequently affected with MIA-induced neurodevelopmental outcomes than females. Consistent with this notion, a general trend toward the heightened acute inflammation and elevated cytokine levels was shown in murine male vs. female placentas, especially for the abundant cytokines, such as IL-6, upon maternal stimulation with LPS [344].

Considering all of the aforementioned, it may be concluded that the maternal immune activity in pregnancy may potentially affect the offspring development, cognition, and behavior, through mechanisms including, at least partly, IL-6 and IL-8 signaling. As therapeutic interventions that significantly alter the prenatal environment and systemic non-specific immunomodulatory agents are not likely candidates in pregnant humans, focusing on eliminating the risk of maternal infection remains the main prevention strategy to reduce the incidence of neurodevelopmental abnormalities.

Last, but not least important, it must be taken into consideration that in humans, most gestational infections do not lead to overt neurological/psychiatric disease in the offspring, despite the strong evidence that the stimulation of the maternal immune response during gestation has a potential for profound effects on the offspring neurodevelopment [326,345]. In fact, it may be assumed that MIA acts more as a “disease primer”, by establishing a susceptible neuroanatomical/neurophysiological setting which, coupled with a permissive genetic background, may increase sensitivity to the disrupting effects of postnatal stressors and ultimately result in pathological behaviors and functions later in life [346]. This is reasonable, considering the multitude of highly diverse factors that contri-bute to neurodevelopment, and the multifactorial etiology and complex pathogenesis of neuropsychiatric and developmental diseases [347].

## 4. Conclusions

According to the reviewed literature, IL-6 and IL-8 clearly play multiple functional roles in pregnancy physiology. They appear to contribute to the establishment and maintenance of pregnancy by mediating uterine receptivity, trophoblast function at the implantation site and parturition, the immune-endocrine interactions at the feto-maternal interface and other processes. The hereby presented body of evidence also indicates that a dysregulated IL-6/IL-8 expression, either at the feto-maternal interface or systemically, may contribute to the development of various gestational complications. Therefore, it appears that targeting the IL-6/IL-8 pathways may rescue some pregnancy trajectories and prevent or ameliorate sequelae. Animal models and empirical data suggest several preventive/therapeutic strategies which, directly or indirectly, affect the IL-6/IL-8 production/function. Classic anti-inflammatory drugs, both steroid [348,349,350] and non-steroid [350,351], the application of anti-IL-6 or anti-IL-6R mAbs [352,353,354,355], or anti-inflammatory cytokines [356,357], dietary interventions [358,359,360], the use of probiotics [361,362] or vitamin D [363], microbiota transplants [281,364], and other immunomodulatory interventions have been examined in the context of gestational complications with some success. However, considering that (i) interference with the prenatal inflammatory/immune environment may lead to devastating consequences [365], and (ii) due to complex ethical issues pregnant women are traditionally excluded from clinical trials [366], novel immunomodulatory treatments require an extensive evaluation on both a scientific and ethical basis before being routinely implemented in a clinical setting. Thus, increasing awareness and optimizing prevention by correcting modifiable risk factors for gestational complications associated with dysregulated inflammatory/immune responses, should remain one of the main strategies in prenatal care.

Finally, one of the limitations of the present review for a successful translation to the clinical level, is its focus on IL-6 and IL-8 only. This reductionist approach enables a better overview of the role of IL-6 and IL-8 in pregnancy-related processes, but is insufficient to draw accurate conclusions about the inflammatory status. This is reasonable considering the complex cytokine networks that underlie these processes and the dynamic relationship between the pro- and anti-inflammatory factors over the course of inflammation.

The emerging advances in biomedical research that enable the computer modeling of data and the new insights into the fields of genomics, epigenetics, proteomics, metagenomics (the microbiome), and metabolomics, and will hopefully improve our understanding of the molecular mechanisms of pregnancy and its possible complications. Such an understanding could be employed to tailor the diagnostic/therapeutic strategies for a more personalized healthcare. Bridging the gaps in knowledge identified herein, could contribute to optimizing the current practices to improve pregnancy outcomes.

## Figures and Tables

**Table 1:** Involvement of IL-6 and IL-8 in a healthy pregnancy.

Process	Role	Cytokine	Reference
**Pregnancy establishment**	Endometrial receptivity and implantation	**IL-6**	[14,28]
		**IL-8**	[48,49]
	Stimulation of human embryo blastulation and blastocyst hatching in vitro	**IL-6**	[33]
	Potential indicator of human embryo developmental potential in ARTs *	**IL-8**	[51]
	Regulation of trophoblast invasion and migration in vitro	**IL-6**	[21,43,44]
		**IL-8**	[23,24,26,53,55]
	Spiral artery remodeling	**IL-6**	[45]
		**IL-8**	[45,54]
**Parturition**	Key gene triggering specific mechanisms in different gestational tissues leading to labor onset	***IL6***	[56]
	Most upregulated gene in the myometrium and cervix with the onset of labor	***CXCL8***	[57]
	Stimulation of prostaglandin synthesis by decidual cells and chorioamniotic membranes	**IL-6**	[58,59]
	Stimulation of oxytocin secretion and oxytocin receptors’ expression on MSMCs *	**IL-6**	[60,61]
	Cervical remodeling and rupture of the gestational membranes	**IL-8**	[62,63]
	**Circulating concentrations**		
**Pregnancy**	Increased over the course of pregnancy	**IL-6**	[64,65,66,67]
	No difference between trimesters	**IL-6**	[68,69,70,71,72]
	Decreased over the course of pregnancy	**IL-6**	[73,74]
		**IL-8**	[75]
	U shape (decreased between the 1st and 2nd trimesters and increased between the 2nd and 3rd)	**IL-8**	[68,69,70,76]

* ARTs—artificial reproductive technologies; MSMCs—myometrial smooth muscle cells.

## Data Availability

Not applicable.
